# Temporal Structuring as Self-Discipline: Managing time in the budgeting process

**DOI:** 10.1177/01708406221137840

**Published:** 2022-12-09

**Authors:** Ferdinand Kunzl, Martin Messner

**Affiliations:** University of Innsbruck, Austria; University of Innsbruck, Austria

**Keywords:** budgeting, entrainment, temporal self-discipline, temporal structuring, temporality

## Abstract

We examine how actors engage in *temporal self-discipline* so as to achieve entrainment of a practice to temporal norms. Temporal self-discipline is about imposing self-created temporal structures on one’s future behaviour and goes along with the (re-)production of a time-conscious self. Based on our fieldwork, we show how such self-discipline materializes both in the form of a very detailed temporal plan and in spaces for coordination to ensure sticking to this plan. We demonstrate that practising temporal self-discipline provides accountants with a sense of control over the budgeting process – a way to achieve ‘controlled’ entrainment to the temporal norm. We also show how temporal disruptions may challenge controlled entrainment, forcing actors into a passive mode of reaction and potential deviation from their intended plan.

## Introduction

There is an important temporal dimension to the management of organizations, as is visible in a variety of temporal structures, such as daily working hours, reporting deadlines or meeting rhythms (Feldman, Reid, & Mazmanian, 2020; Orlikowski & Yates, 2002). Several studies have examined the politics around such temporal structures and their potentially contested nature, offering interesting insights into how actors craft, challenge or negotiate such structures (Granqvist & Gustafsson, 2016; McGivern et al., 2018; Oborn & Barrett, 2021; Reinecke & Ansari, 2014).

In many contexts, however, actors will take existing temporal structures for granted, not least if these are firmly institutionalized within the environment (e.g. academic year) or organization (e.g. opening hours of a business). Rather than challenging or negotiating such structures, actors’ efforts are then targeted at ‘entraining’ to them (Ancona & Chong, 1996). Such entrainment has sometimes been portrayed as a somewhat automatic or uneventful response to an external demand (Ancona & Chong, 1996; see Granqvist & Gustafsson, 2016) and therefore as lacking active agency, at least when compared to the more activist forms of contesting, negotiating or creating temporal structures (e.g. Blagoev & Schreyögg, 2019; Granqvist & Gustafsson, 2016; McGivern et al., 2018; Reinecke & Ansari, 2014).

We challenge such an understanding of entrainment in this paper and suggest that entrainment to a given temporal norm is not necessarily unproblematic or uneventful. This is true not only for situations of high uncertainty (e.g. Geiger, Danner-Schröder, & Kremser, 2021; Kremser & Blagoev, 2021; Patriotta & Gruber, 2015), but also in settings where temporal norms are stable, but entrainment to them requires considerable efforts and agency. We use an empirical study to examine one such setting, where actors deal with the challenging task of entraining to deadlines and where they resort to *temporal self-discipline* (see Foucault, 1977; Grey, 1994; Roberts, Sanderson, Barker, & Hendry, 2006) – a form of agency whereby actors craft, and subject themselves to, additional temporal structures in order to ensure entrainment.

Such temporal self-discipline is of wide empirical relevance, since many contemporary work settings are characterized by somewhat flexible temporal structures (de Vaujany, Leclerq-Vandelannoitte, Munro, Nama, & Holt, 2021). Organizations expect employees to entrain their activities to these temporal structures (e.g. achieving a particular performance by then), but leave discretion to their employees as to how to use their time within these intervals. This constitutes a ‘governing’ form of control (Foucault, 1982; Miller & Rose, 1990), which makes the individual responsible for their conduct and, not least, for using their time ‘wisely’ (Blue, 2019; Feldman et al., 2020; Portschy, 2020).

Our paper examines how, in such a setting, *temporal self-discipline can emerge; how it materializes; and what power dynamics it is subject to*. To address these questions, we draw upon a qualitative study of a budgeting process in a manufacturing plant. We study accountants’ collective efforts to temporally structure the budgeting process in light of a corporate deadline. We identify two conditions (dominant temporal norm; complexity of the practice) that motivate the emergence of temporal self-discipline. Temporal self-discipline materializes both in the form of a very detailed temporal plan (‘blueprint’) and in ‘spaces for coordination’ to monitor performance according to this plan. We demonstrate that practising temporal self-discipline provides accountants with a sense of control over the budgeting process – a way to achieve ‘controlled’ entrainment to the temporal norm. We also show, however, how temporal disruptions may challenge controlled entrainment, forcing actors into a passive mode of reaction and potential deviation from their intended plan.

Overall, our paper contributes to a better understanding of how actors achieve entrainment to temporal norms or events (Geiger et al., 2021; Kremser & Blagoev, 2021; Patriotta & Gruber, 2015). We show how temporal autonomy can go along with actors creating restrictive temporal structures for their future behaviour. Hence, somewhat paradoxically, the restriction of flexibility may be the very expression of one’s temporal autonomy (Mazmanian, Orlikowski, & Yates, 2013). Moreover, the paper improves our understanding of the temporal dimension of self-discipline, which not only is temporal in the fundamental sense of being anticipatory (Costas & Grey, 2014; Grey, 1994; Hoy, 2009; Portschy, 2020), but may also appear in the form of a detailed temporal structuring of one’s future behaviour, potentially contributing to a particular type of subjectivity.

## Temporal Structuring, Entrainment and Autonomy

Prior research shows that organizational actors may have different degrees of autonomy over the temporal dimension of their work (Feldman et al., 2020; Orlikowski & Yates, 2002).

In some cases, actors exercise a high degree of autonomy insofar as they can *define or change* the temporal structures (norms) that guide their own and others’ activities. This is the case when strong external ‘*Zeitgebers*’ (Ancona & Chong, 1996) are absent. Granqvist and Gustafsson (2016), for instance, show how university managers were able to shape the timeline of a wider reform project through different forms of ‘temporal institutional work’. Such creative temporal structuring may also happen more covertly, as in the case of consultants who inscribe their preferred temporal norms into project documents and thereby align clients with these temporal structures (McGivern et al., 2018). In yet other cases, actors negotiate temporal structures more democratically, engaging in what Reinecke and Ansari (2014) call ‘temporal brokerage’.

In contrast, other temporal structures are difficult to change or challenge, and actors will have to entrain their activities to these Zeitgebers (Ancona & Chong, 1996). Even then, however, they will seek to maintain some level of control over *how* they achieve entrainment (Lupu & Rokka, 2022). Research shows that they do so not least by following particular routines or protocols, which they then mindfully adapt to emerging circumstances. For instance, Patriotta and Gruber (2015) show how newsmakers would largely pre-plan the daily news broadcast, being prepared to change plans at short notice when unexpected news emerge. They succeed in doing so by quickly typifying news according to a set of routine questions. Similarly, Geiger and colleagues (2021, p. 235) show how firefighters, when confronted with an emergency, enact trained routines which allow them ‘to control the situation’ rather than reacting ‘hastily and without proper preparation’. They would use cues from the situation to flexibly adapt how they enact these routines (e.g. with what pace). Kremser and Blagoev (2021) show how consultants perform their work by relying on a set of routines, but would re-prioritize these depending on emergent contingencies.

It is interesting that the above-mentioned studies characterize their empirical settings by pointing to the high level of uncertainty and unpredictability in these settings, which render complete pre-planning of tasks impossible. Temporal structuring is therefore more creative and dynamic in these settings than pre-defining a ‘formal schedule that works for everyone’ (Kremser & Blagoev, 2021, p. 339). We very much sympathize with the general thrust of these studies and their interest in how actors seek to maintain temporal control despite an uncertain course of events. Yet, we also suggest that ‘staying in control’ may likewise be challenging in those cases where actors face a stable external Zeitgeber and can largely pre-plan their activities. This is because significant *effort* may be needed to ensure entrainment to external temporal demands (such as deadlines). Actors may engage in rather detailed and disciplined forms of proactive temporal structuring, reflecting their desire to stay in control over their use of time (Lupu & Rokka, 2022).

We suggest theorizing such efforts as an expression of *temporal self-discipline* (Foucault, 1977, 1982; Grey, 1994; Roberts et al., 2006) – a form of temporal structuring in which actors subject themselves to self-created temporal structures so as to achieve ‘controlled’ entrainment to externally imposed temporal norms. By exercising self-discipline, actors maintain a strong level of control over the temporal structure of their work. Unforeseen events may also emerge in such a case however, challenging actors’ sense of ‘being in control’ more strongly than in settings where actors are used to reckon with the unexpected (Geiger et al., 2021; Patriotta & Gruber, 2015). Indeed, we see an interesting tension between one’s dedication to a self-defined temporal structure, on the one hand, and the possible disruptions of such a structure, on the other hand.

## Temporal Structuring and Self-Discipline

### Self-discipline

Discipline is a set of techniques that seek to control the details of individual behaviour (Foucault, 1977). It works through spatial mechanisms, such as the enclosure and partitioning of space, which render visible individuals and their behaviour. It also relies on the control of activity in time, as realized through timetables and detailed specifications for how to perform activities. Disciplinary power is at work in a range of organizational practices that exercise control through constant surveillance, routinization and examination (e.g. Knights & Willmott, 1989; Sewell & Wilkinson, 1992; Townley, 1993).

Contemporary forms of organizational control are not primarily based on disciplinary power, however. In his later work, Foucault (1982) shifted his concern from disciplinary practices, where the individual’s freedom is constrained, towards those forms of control that rely on, and appeal to, individuals’ freedom (Munro, 2012). Indeed, today’s organizations typically do not constantly monitor employees or subject them to detailed action controls. Rather, control operates to an important extent by making people accountable for outcomes or by appealing to aspirational identities, career prospects or corporate values (Burchell, Gordon, & Miller, 1991; Costas & Grey, 2014; Grey, 1994; McKinlay & Taylor, 2014; Miller & O’Leary, 1994; Miller & Rose, 1990). Such forms of control require individuals to make their own decisions about specific actions and to become responsible stewards of themselves. In such a regime, employees are ‘governed’ rather than directly ‘disciplined’.^1^

In governmentality regimes, individuals may however engage in disciplinary practices themselves, in order to achieve expected outcomes (Roberts et al., 2006). Such *self*-discipline relies on some of the same mechanisms that Foucault highlights in his study of the disciplinary apparatus: managers or employees self-assess against norms and standards, they create visibility over their own actions, and they subject themselves to particular routines and ‘exercises’ that allow them achieve external expectations (Covaleski, Dirsmith, Heian, & Samuel, 1998; Grey, 1994; Lambert & Pezet, 2011; Roberts et al., 2006). The resulting practice is hence a combination of hierarchical control and self-control, arguably constituting a ‘more productive and economical form of management control than disciplinary power’ (Grey, 1994, p. 495).

The interplay between hierarchical control and self-discipline is not least visible in the temporal structuring of work, as explained next.

### Temporal self-discipline

One of the mechanisms through which discipline operates is that of temporal structuring, as realized by means of imposed schedules, timetables or mandatory routines (Foucault, 1977). In a work context, such a form of temporal control is evident in the detailed task scheduling on the production line (e.g. Perrucci & MacDermid, 2007; Taylor, 1911), but also in mandated routines of time-recording for knowledge workers (e.g. Anderson-Gough, Grey, & Robson, 2001; Brown & Lewis, 2011). The more tightly knit such imposed temporal structures are, the less flexibility employees have over the use of their time (Feldman et al., 2020) and the more organizations can ‘discipline’ employees into adopting a particular temporal behaviour.^2^

In a governmentality regime, control of time is less detailed and more indirect in nature. Performance is measured and assessed at aggregate time intervals (e.g. monthly, quarterly), and employees have flexibility not only in terms of what they do but also when to do it. Such ‘empowerment’ goes along with the expectation to regard time as a valuable resource (Blue, 2019; Feldman et al., 2020; Portschy, 2020). This requires employees to temporally structure their work in an autonomous manner, and this is where temporal self-discipline may emerge.

Temporal self-discipline may build upon the same mechanisms of temporal control that are found in disciplinary settings (timetables; detailed measurement of time; regular routines; etc.). In the case of self-discipline, however, these forms of temporal structuring are self-imposed, individually or collectively. They are means to achieve entrainment to aggregate temporal expectations (e.g. performance targets at the end of the year). Temporal self-discipline is not about passively reproducing existing temporal norms, but about adopting ownership of achieving entrainment to given norms. It is therefore an expression of control over one’s use of time (Feldman et al., 2020; Lupu & Rokka, 2022).

Yet, temporal self-discipline is not the opposite of temporal power that others have over one’s time. Rather, it is often *because* of the power of others that such forms of self-discipline emerge in the first place, i.e. in order to comply with externally defined temporal demands. Accordingly, self-discipline is also susceptible to being overruled or disrupted by interventions from those who have defined these demands and hold hierarchical power. Hence, while temporal self-discipline is an expression of autonomy over one’s time-use, such autonomy may prove fragile (Mazmanian et al., 2013).

We examine the emergence of temporal self-discipline as well as the power dynamics rendering it fragile in an empirical setting where temporal self-discipline becomes particularly salient.

## Empirical Context of our Study

We investigate temporal self-discipline in the context of the annual budgeting process in a production site. Budgetary control is a prime example for a governing form of power. As ‘a norm of employee performance and self-appraisal’ (Robson, 1992, p. 700), budgets induce self-disciplined behaviour on the part of managers who adopt responsibility for meeting budgetary goals. While this self-disciplining role of budgets is well documented in the literature (e.g. Hopper & Macintosh, 1993; Miller, 2001; Miller & O’Leary, 1987), its temporal dimension is much less explored (Becker & Messner, 2013).

We suggest that this temporal dimension of self-discipline is particularly pronounced for actors in charge of managing the budgeting process. Accountants typically assume this role of a ‘process owner’. They are responsible for coordinating budget-inputs of various organizational actors and for ensuring that budgets are finalized on time. Budgeting constitutes an important activity within the work of accountants; performing the budgeting process well is therefore also high on their agenda (Mouritsen, 1996). Such ‘performance’ is not least a temporal one, as budgeting is embedded in a rigid timeframe, with the financial year serving as its Zeitgeber (Ancona & Chong, 1996). Accountants are generally used to thinking in terms of the financial year and their profession can be regarded as highly ‘temporalized’ (Anderson-Gough et al., 2001). In a Foucauldian sense, the budgeting process then contributes to reproduce this temporal dimension of their subjectivity. This does not mean, however, that achieving entrainment is automatic or effortless for accountants. Precisely how they deal with the need to entrain the budgeting process in temporal terms is the focus of our study, from which we seek to generate insights into the dynamics of temporal self-discipline.

## Research Methods

### Data collection process

We collected qualitative data in the largest production site (DrugSite) of one division (DrugDivision) of a multinational group (DrugGroup) within the pharmaceuticals industry.^3^ DrugGroup ranks among the ten largest pharmaceutical firms worldwide in terms of revenues. At the time of our research, DrugDivision accounted for about one-fifth of these revenues. DrugSite was the largest development and production site within DrugDivision.

Our data collection encompassed three phases (see Appendix 1).

#### Phase 1: Observing and exploring

The exploratory and inductive first phase of data collection helped us become familiar with the case organization (e.g. Gioia, Corley, & Hamilton, 2013). Initially, one researcher spent one and a half days at DrugSite and was provided an extensive overview of the organization and its budgeting process. Shortly thereafter, DrugSite’s accountants initiated a formal workshop-based project to optimize their local budgeting process. We attended six workshops, taking notes that we later transcribed.^4^ One of the researchers spent various days in the department, having informal conversations with accountants, discussing the optimization project with them and observing team meetings. These observations sensitized us for accountants’ perspective on the budgeting process and helped us develop our set of questions for the interviews (see below). Various internal documents complemented our understanding.

#### Phase 2: Semi-structured interviews

We conducted 21 semi-structured interviews, starting with DrugSite’s accountants whom we already knew personally from the first phase of our fieldwork, and then moving to other interview partners. By the end of this interview round, we had interviewed most of DrugSite’s accountants who were involved in budgeting.

In a second round of interviews, we interviewed other actors with whom local accountants would interact during the budgeting process, such as operational managers and divisional accountants at the headquarters. Interviewees were chosen by asking DrugSite’s accountants for their most frequent and most important counterparts outside the department during the budgeting process.

Interviews lasted for approximately 60 minutes, were audio-recorded and transcribed verbatim. Except for two, all interviews were conducted by both researchers. Interviews were conducted (and analysed) in the language of the interviewees and quotes selected for the empirical part of the paper were translated into English.

#### Phase 3: Follow-up data collection

The final phase of data collection involved a meeting with the head of the accounting department and DrugSite’s CFO to discuss our observations. We also conducted two more interviews with the accountant responsible for coordinating the budgeting process at DrugSite and her predecessor.

### Data analysis and theorization

**Figure 1.**
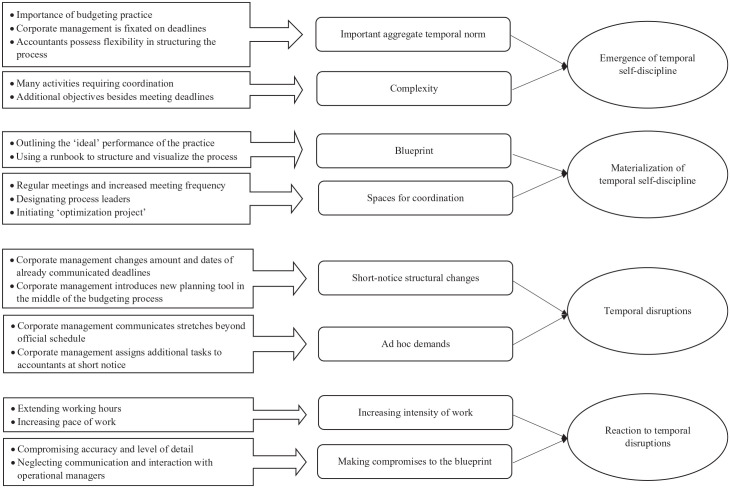
Data structure.

Having started with a broad interest in the management of the budgeting process, we incrementally developed a temporal structuring focus. Data analysis was an ongoing process, including various iterations of coding during and after data collection (Gioia et al., 2013; Langley, 1999). Figure 1 displays the data structure resulting from our coding (Gioia et al., 2013). The first-order concepts on the very left represent codes derived directly from the empirical material; the second-order concepts combine these into more abstract categories, which are further aggregated into overarching themes.

We started analysing our data by summarizing early observations in a case narrative. As we realized that accountants’ narrations were rich in references to temporality, our subsequent coding was guided by literature on temporal structuring and entrainment (Ancona & Chong, 1996; Orlikowski & Yates, 2002). We realized that accountants faced externally imposed temporal structures (in the form of deadlines) in the budgeting process to which they entrained their own activities. More interestingly, however, they imposed upon themselves additional temporal structures in order to ensure compliance with the given temporal norms. This strategy of ‘disciplining’ oneself to a particular course of action was different from the active structuring efforts described in extant literature (e.g. Granqvist & Gustafsson, 2016; Kremser & Blagoev, 2021; Patriotta & Gruber, 2015).

To make systematic sense of this observation, we turned to literature on (self-)discipline (Foucault, 1977, 1982; Grey, 1994; Roberts et al., 2006). This led us to theorize accountants’ efforts as an expression of *temporal self-discipline*. Such self-discipline materialized in accountants’ efforts to anticipate the process and create a mental and material plan for it – which we came to call a *blueprint*. We also realized that accountants would not only rely on this blueprint, but would create various *spaces for coordination* to remind themselves of the blueprint and facilitate entrainment to the temporal norm. In addition, we reflected upon, and coded for, the conditions that would trigger or facilitate such self-discipline, realizing that an interplay between an *important aggregate norm* and the *complexity of the practice* accounted for this.

Moreover, our analysis of the data revealed that accountants were sometimes not able to follow their self-devised temporal structures. We focused on identifying the conditions under which this was the case and could associate such deviations primarily with hierarchical interventions that accountants perceived as *disruptive*. Finally, we coded for accountants’ *reactions* to such disruptions (increasing work intensity; making compromises).

## Empirical Findings

### The emergence of accountants’ temporal self-discipline

As mentioned above, accountants are used to thinking in terms of the financial year and many of their activities are entrained to this temporal rhythm (Anderson-Gough et al., 2001). The budgeting process is one manifestation of such a need for entrainment, insofar as budgets should be finished before the new financial year starts. Yet, how precisely the (temporal) demands for budgeting look like, and to which extent accountants react to them with temporal self-discipline, is an empirical question. We therefore first examine the organizational conditions that made DrugSite’s accountants engage in temporal self-discipline.

#### The importance of an aggregate temporal norm

The annual budget assumed a very prominent role within DrugGroup. When asked about it, employees across different functions frequently cited a statement by a previous CEO that reflected the incontestability of the budget: ‘A target is a target is a target’.^5^ Accountants confirmed that such budget emphasis was deeply rooted in DrugGroup’s strong shareholder orientation. There was high awareness that the organization was ‘obligated towards its investors to communicate growth’ (Eric), which also translated into ‘ambitious business objectives’ (Thomas).

Shareholder orientation also had a temporal dimension, as the financial year acted as the unquestioned Zeitgeber (Ancona & Chong, 1996) for the internal budgeting process. To ensure entrainment to it, DrugGroup imposed strict submission deadlines on its divisions, which were issued in a planning calendar at the beginning of each planning year. DrugGroup was known for being ‘very fixated on deadlines’ (Ethan) more generally and accountants perceived budgeting deadlines in particular as a strong temporal norm towards which they had to entrain their activities:

That’s already anchored in our thinking, that it’s basically taken for granted. It is no longer questioned. [. . .] An official, globally communicated deadline will be kept. (Brian)

The temporal norm was of an *aggregate* nature insofar as DrugGroup temporally orchestrated the process with a few deadlines only, rather than specifying it in each and every detail. Indeed, apart from the deadlines, accountants perceived considerable flexibility in managing the budgeting process:

Basically, there are three milestones, I would say. First, there is the final submission including new production costs, that’s in November. Second, the first submission of the [budget] in September. [Third], the [budget] presentation with different business sectors, that’s in August [. . .]. These are the dates that we have to meet . . . everything else is more flexible. (Arnold)

The aggregate nature of the temporal norm empowered and mandated accountants to act autonomously between the deadlines. They were not disciplined by others into a tightly knit temporal structure (Foucault, 1977), but were rather governed through aggregate temporal norms (Foucault, 1982; Miller & Rose, 1990). They were given the freedom – and responsibility – to manage the budgeting process in ways to achieve entrainment to imposed deadlines. Such responsibility was also reflected in accountants’ self-understanding as ‘process owners’; they saw themselves responsible – and inherently needed – for holding ‘the whole thing together’:

Well, we are very strongly required here. We have to be very active in collecting everything and manage the whole thing from [the accounting department], otherwise it would immediately get out of hand. (Thomas)

The importance of reaching the deadlines was therefore not just seen as an ‘external’ demand; accountants had internalized the need to manage the budgeting process and regarded it as part of their professional role. Being a good accountant meant, inter alia, being able to hold the budgeting process together. Therefore, the need to complete budgeting on time was not a ‘necessary evil’, but also an opportunity for the accountants to prove their ‘value added’ and to ‘realise their own . . . aspirations’ (Grey, 1994, p. 488) as process owners.

An important and aggregate temporal norm is not sufficient for triggering temporal self-discipline however. For if the focal practice is rather simple, there would seem to be little need to invest in elaborate temporal structuring efforts. As shown next, it was the *complexity* of the budgeting process that created the need for such structuring.

#### The perceived complexity of budgeting

Budgeting in DrugSite was a complex practice in two respects. First, it consisted of a *manifold of interdependent activities*. Second, *other objectives* over and above meeting deadlines had to be accounted for. These two dimensions of complexity rendered entrainment a challenging task for accountants, and temporal self-discipline was their answer to this challenge.

Establishing a budget involved a variety of different steps, most of which revolved around the estimation of different types of costs in various business areas. These activities had to be coordinated in terms of who should do them when. Often, one step (such as calculating costs in one area) was a precondition for another step (such as allocating these costs to products). Accountants sensed that failing to plan this sequence correctly and to stick to this plan would endanger the whole process: ‘If one step does not happen, then the next [person] cannot do their work’ (Arnold).

Hence, accountants meticulously planned the different activities and their sequence in advance (as we will show in more detail below). Note that complexity required *self*-discipline, as accountants were responsible for managing the process and as most of the activities within this process were not pre-defined by someone else and were performed by accountants themselves. However, part of the complexity emerged because of the involvement of other actors in the organization. For instance, cost center managers provided cost estimations for their cost centers, and the HR department estimated personnel costs based on FTE numbers communicated to them. This input also had to be coordinated, such that self-discipline extended into the temporal control of other actors’ activities. The awareness of the need to plan and coordinate the input of other actors, in turn, reinforced accountants’ self-understanding as the ‘process managers’ who were needed to keep the process together:

It’s not that it comes out intrinsically from [managers], but it is the [accounting] department that always holds the whole thing together like a bracket and whoops [the process]. (Thomas)

We see the adoption of such a role as a ‘process manager’ as a fundamental part of accountants’ temporal self-discipline. For it implies not only managing the involvement of other actors, but also to take responsibility for the temporal structure of the budgeting process as a whole.

Accountants faced complexity also in another respect: Meeting deadlines was not the only objective accountants associated with the budgeting practice. In addition, they aimed for completing the budget in *a certain form*. They repeatedly emphasized how important it was for them to produce budget numbers that would be ‘accurate’.^6^ Such accuracy comprised two aspects: first, calculations had to be computationally correct and adhere to accounting standards; providing wrong numbers was out of question for accountants, who considered themselves producers of truthful numbers (see Lambert & Pezet, 2011):

There is no possibility that [the accounting department] submits a wrong number. That’s just a no-go. If our numbers are not correct, which numbers are correct? (Arnold)

Second, accountants spoke of ‘accuracy’ also in terms of producing a budget that was both challenging and realistically achievable – an aspiration that is commonly associated with ‘good’ budgets (Merchant & Manzoni, 1989). Realizing this ambition required accountants to reduce budgetary slack by challenging managers:

Our task in this regard is actually to really challenge [managers] and to say: ‘Okay, [costs are] still too high, we’ll have to look at that’, and so on. (Peter)

Extracting more performance from the business reflected accountants’ acceptance of the norm of creating shareholder value. Anticipating the expectations of shareholders and corporate management, accountants even came to see this norm as being self-imposed, such as when Brian suggested: ‘That’s driven by us, that we really must provide a good plan’. Such internalization of external expectations demonstrates accountants’ self-disciplining behaviour (Grey, 1994; Roberts et al., 2006). Their aspirations to produce a good budget, in turn, had implications for the need to temporally structure the budgeting process. For while accountants were confident that they could process all activities accurately, they stressed that ‘this takes time’ (Arnold). Striving for a ‘good’ budget required discussions with managers and agreeing on numbers that could be submitted in good conscience. This increased the complexity of the practice in terms of having to accommodate additional steps in the process. Time was therefore considered a highly scarce resource (Blue, 2019; Portschy, 2020):

And time is exactly what we lack [. . .] during [the budgeting] process [. . .]. We have everything but time. (Arnold)

To summarize, the aggregate nature of deadlines provided accountants with the freedom and responsibility to self-manage their activities (Miller & Rose, 1990). The complexity of the practice rendered entrainment to the deadlines challenging, which is why self-managing became a matter of adopting temporal self-discipline.

### The materialization of accountants’ temporal self-discipline

We observed two forms in which temporal self-discipline materialized: a *blueprint version* of the practice and *spaces for coordination*. The blueprint provided a ‘script’ (Roberts et al., 2006) against which accountants could surveil their own performance; spaces for coordination were used to remind oneself of the blueprint, enforce it, and possibly make small adjustments to it over the course of the process.

#### Creating a blueprint

In order to ascertain what was required during the budgeting process, accountants perceived the creation of a *blueprint version* of the practice as indispensable. Their efforts were thereby primarily oriented towards the deadlines as specified by corporate management:

We wait until the [calendar] gets published. [. . .] It specifies the first submission date. And this is basically the starting point for us [. . .]. Because then we basically calculate [backwards], so as to know when we approximately have to start so as to have all the data ready until the first submission. (Andy)

Accountants used the corporate calendar for orientation, nourishing it with the routine and predictable aspects of the process. This enabled them to estimate the point in time required to start with budgeting and to temporally pre-structure the practice. Such pre-structuring implied defining the length and sequence of different activities, incorporating (small) time buffers to ‘have two days or so to prepare the files that we have to submit, to polish them and to check again the numbers’ (Kate). The blueprint thus constituted the idealized outline of the budgeting process based on the information available at the beginning of the process.

Creating a blueprint raised accountants’ awareness of the process and their respective role in it; ideally, everyone should become aware ‘that until that day, until that week, until that calendar week, this and that [activity] must be done’ (Thomas). Arnold highlighted the goal ‘to have a process, a clearly defined process so that everyone knows, until then I have to do this and if it doesn’t work out, I have to escalate it’. The blueprint was therefore a form of disciplining oneself in terms of when to do what. Accountants felt that they required ‘a tightly streamlined schedule’ that ‘just like a cogwheel system meshes in an exactly timed manner’ (Thomas).

To further increase the usability of the blueprint for self-disciplining, there was even an effort to formalize it into a shared document that all accountants could access and edit. This so-called ‘runbook’ meticulously listed all necessary steps, the responsible person, the respective business unit, start and due dates for each activity, priorities, the status of activities, and indicated other deadlines that had to be considered for the respective activity. For the preparation of DrugSite’s budgeted income statement, the runbook comprised 173 steps to be carried out. The idea was that every accountant should update the runbook whenever completing a task, thus allowing constant surveillance of the collective performance. While the runbook was used during team meetings, the idea of individually updating it was ‘never really lived’ (Ethan). Nonetheless, it was an expression of accountants’ self-disciplined approach to temporally pre-structure the practice.

Overall, the blueprint helped accountants to craft and visualize their ‘ideal’ performance of the practice before actually starting with it, accounting for everything it would take to achieve entrainment in a controlled manner. It manifested their deliberations about *what* needed to be done, *when* the constitutive activities had to be performed, and *how* these were to be carried out. It thus provided accountants with a detailed script (Roberts et al., 2006) that kept their self-created temporal structures and that could be used to discipline their performance during the process.

#### Creating spaces for coordination

Spaces for coordination complemented the blueprint. They had the purpose of enforcing accountants’ adherence to the blueprint and allowing them to adjust it when needed, thus accounting for aspects that could not be anticipated. Our empirical data evidence two spaces for coordination. First, so-called process leaders were installed. Second, accountants increased the frequency of team meetings during the most intense phases of the process to foster communication.

Governmentality literature suggests that particular rationalities and ideas are not only operationalized through specific technologies, such as the planning calendar or the runbook (Miller & Rose, 1990), but also through the work of intermediaries who, like a pastor (Foucault, 2007), guide other actors in their self-disciplined behaviour (Martin & Waring, 2018). Such intermediaries can thus be seen as a materialization of (collective) self-discipline and, in our case, their role was particularly focused on the temporal dimension of the budgeting process.

While Arnold coordinated DrugSite’s budgeting process on the departmental level, one accountant within each team was installed as process leader. They kept an eye on the overall process, informed colleagues about upcoming activities and internal deadlines, and intervened when delays from the blueprint occurred. Process leaders emphasized the importance of deadlines and the need to submit ‘good’ budgets on time:

I coordinate the whole thing [for my team] so that we can provide good data quality on time. [. . .] I am simply responsible that all steps are made and that we can submit on time. Meaning, everyone has his [sic] duties within the team and I just keep track if they are done in good time or not. (Andy)

Ethan stated that this role was ‘primarily about coordination and timing, to have a look at [when] the deadlines actually are at the end and when we must be finished’. Julia highlighted the importance of ‘having one central person’ responsible for sending ‘out a reminder [. . .] two days, three days before [a deadline]: “Watch out, don’t forget this”.’ Hence, process leaders acted in the name of the blueprint; they were co-responsible for making sure that other accountants performed according to these temporal structures and thus in a disciplined manner. Moreover, acting according to their temporal self-discipline sometimes involved reminding other actors (such as operational managers) to submit their input, if these ‘did not comply with the dates or did not deliver the data in the needed quality’ (Thomas).

A second measure for making sure that accountants would entrain to their self-created temporal structures was to increase the frequency of meetings within the department during the most intense phases of the process, i.e. during times at which entrainment was particularly at risk. This should provide accountants with a regular platform for updating and sensitizing each other for possible deviations from the blueprint. Meetings also helped process leaders to keep an overview of the ongoing developments.

[W]e always had a weekly meeting, which helped us internally, locally a lot [. . .]. And in the meetings on Mondays I basically heard certain things for the first time. . . [For example,] that [. . .] one team has to submit the day after tomorrow or something. (Julia)

Similarly, within the different teams, accountants also coordinated their activities by meeting more frequently, supporting and reminding each other of acting according to the blueprint: ‘We sit together: “Okay, which tasks are still to complete?” We really write down a list and say: “Who is doing what and until when?”’ (Kate).

Moreover, meetings created transparency within the accounting department that further contributed to enforcing discipline. Teamwork, more generally, may create strong disciplinary effects as team members monitor each other’s behaviour and enforce discipline on each other (Barker, 1993; Sewell, 1998). Reporting to each other about their respective status implied that accountants had to admit when they were ‘lagging behind’, exposing themselves as the ones jeopardizing the process due to personal undisciplined behaviours. Observations during a team meeting held after one interim submission of the budget are particularly insightful in this regard. Arnold voiced his disappointment with some accountants who had left the office without having checked their numbers so that their undisciplined behaviour had to be compensated by colleagues:

Because at the [. . .] submission date [. . .] we went home at 1 [o’clock] in the morning with five people. Because we didn’t cope with it. Because no one carried responsibility. [One team member] drove home without having checked his numbers! (Arnold)

Openly pillorying some of the accountants during a meeting thus served the purpose to (publicly) reaffirm accountants’ own expectations towards themselves, emphasizing that undisciplined behaviour could not be accepted and would be sanctioned.

To summarize, accountants’ temporal self-discipline materialized in two ways: a *blueprint* version of the practice and *spaces for coordination*. These measures emphasize how accountants assumed responsibility for organizing the process and exercised (collective) self-discipline (Foucault, 1982). They were tantamount to accountants seeking to remain *in control* of the practice.

### Temporal disruptions

In terms of using the available time, accountants sensed being ‘very much at the limits regarding squeezing the orange dry. Because everything is timed so tightly’ (Thomas). Accordingly, performance along their self-created temporal structures only allowed for minimal deviations. Next, we show how accountants perceived various interventions on the part of corporate managers as unexpected temporal disruptions that put controlled entrainment at risk. Such disruptions made accountants feel they were losing control of the practice and being ‘forced’ to move away from autonomous performance.

#### Short-notice structural changes

One disruption related to corporate management’s tendency to make structural adaptations at short notice. Accountants reported that corporate management sometimes changed ‘fixed’ timelines without considering the broader implications:

[Corporate managers] always say: “It will only be this amount of submissions and . . . it’s never like that. [. . .] In the beginning [. . .] we have a schedule. But this is constantly adjusted. (Rachel)

Additionally, accountants claimed that deadlines were sometimes ‘claimed earlier’ (Vincent). Paula outlined how an important budget meeting was shifted forward without communication: ‘We actually saw it two weeks prior or so: “Oh, that’s on that date”.’ Julia summarized: ‘In the beginning, we were a little bit naïve and trusted the global deadlines.’ Somewhat ironically, while constituting the basis of their temporal pre-structuring, accountants sensed that corporate deadlines could not always be trusted.

Similarly, the implementation of a new planning tool was perceived as another instance of corporate disruptive behaviour. Accountants claimed that the tool had been ‘more or less forced [. . .] on us additionally’ (Peter) by corporate management. They were specifically upset about the fact that it disrupted *their* self-created temporal structures and prevented them from acting upon these. The new tool – respectively, corporate management’s implementation style – was even made responsible for ‘all the chaos [that] came about’ (Vincent) during that budgeting period. Having to comply with the imposed demands to implement the new tool in the middle of the budgeting process was a ‘surprise’ to the accountants, not least because it disrupted their expectations as to how the budgeting process would evolve:

I was especially surprised that you try to remodel such a working [budgeting] process within very, very short time. That surprised me a lot. [. . .] That caused serious difficulties for us. (Peter)

An intervention like this might have well been necessary and beneficial from corporate management’s point of view. To the accountants, however, it represented a disregard for the temporal challenge that the budgeting process already implied for them under normal circumstances.

#### Ad hoc demands

We also observed more ‘everyday’ type of disruptions that were less dramatic, but, taken together, still confronted accountants with challenges in entraining to the final deadline. Accountants voiced particular frustration with ad hoc demands from corporate management, which they felt had no legitimate basis and unnecessarily consumed their precious time. Accountants considered these demands as problematic because they came ‘on top of the actual normal workload’ (Julia) and ‘deterred [us] from the daily, operational business’ (Brian).

Such demands were most evident around the communication of ‘stretches’.^7^ The official corporate planning calendar did not specify the exact dates *when* stretches would be communicated, but accountants knew that they would receive a pre-defined amount of stretches. Their experience allowed them to estimate the approximate time when this would be the case. Accordingly, the handling of stretches was an integral part of accountants’ temporal pre-structuring. As soon as stretches arrived, meetings with operational managers were called to work out joint solutions. It was said to be ‘a tremendous amount of work to represent such a stretch’ in the budget (Andy).

Accountants did not criticize stretches as such; they complained that corporate managers requested *additional* stretches beyond the official schedule:

[This] is an enormous stress for us because every day they strike on a new idea. [. . .] [I]n between the official submission dates, between every submission we surely have two, three [additional] submissions. (Andy)

Accountants even suspected corporate managers were misusing their power and the technical capabilities of the new planning tool for their own purposes, drawing on accountants’ very own temporal resources.

Nevertheless, they always say: ‘Work in the stretch right away.’ In fact, it’s the [corporate managers] who do this so that they don’t have much Excel work to do. Because they really extract their presentations from [the tool]. Now they want to have it all illustrated in the system. [. . .] And they don’t [enter budget adaptations] on their own [. . .]. Instead, they instruct us to do it so that they can push ‘refresh’ again and then they refresh the whole presentation. (Andy)

Andy’s statement illustrates accountants’ perception of no longer feeling (fully) in control of *their* time and, instead, turning into subordinates merely carrying out the orders of corporate managers. Yet, given the importance of the goal of entraining to the deadlines, accountants would undertake additional efforts to compensate for the time that they lost due to such interventions.

### Accountants’ reaction to temporal disruptions

Being confronted with temporal disruptions, accountants first tried to convince corporate managers to refrain from behaviours perceived as unjustified:

We immediately recognize stupid requests and inquiries about what we are supposed to do. [. . .] Our resources are simply extremely limited, so you just have to question many things. (Vincent)

Such pushback or ‘counter conduct’ (Foucault, 2007, p. 196) was an expression of accountants’ wish ‘to be conducted differently. . . through other procedures and methods’ (pp. 194–195). In particular, accountants wanted their autonomy as process managers to be respected and not unduly intervened into. Yet, such resistance was only selective and it also did not always succeed, as corporate managers would often stick to their requests. Our analysis highlights two types of reactions that accountants resorted to in such cases. These reactions implied temporally re-structuring the practice in a way that made accountants incrementally depart from their self-created temporal structures.

#### Increasing work intensity

Even without the occurrence of temporal disruptions, budgeting was a stressful period, as vividly illustrated in the following statement:

Then you just know every day, when this [task] is finished, there’s right away the next topic, the next topic, and. . .. Well, that’s worked off, so to say. [. . .] yes. . . everyone is just stressed. We work a lot. Well, we have partly been to the office until 9 or 10. [. . .] Well, it is like ‘jab jab’ and ‘huh’ and bustling and. . . yes . . . well, it is stress the whole day. (Paula)

Accountants knew that achieving entrainment not least depended on their ability to bear with this stress and to adopt a high-level of self-discipline. When being confronted with additional complications in the form of temporal disruptions, accountants further increased work intensity in order to achieve entrainment. Especially when the scarcity of time escalated, i.e. around submission deadlines, night shifts were the order of the day: ‘Typically, submission is until end-of-business, meaning until midnight – and typically we submit by midnight’ (Julia). Such extra hours were needed not least because corporate management had disrupted the process:

No matter how it comes, it will be done. We will do it. We are very much process-driven. Timelines are . . . well the [budgeting] process will be adhered to. [. . .] The consequence for us means nightshifts [. . .]. You [corporate management] don’t even know that we work during the night so that we can implement what you might not even have thought of. (Arnold)

The increased workload and stress left their mark on accountants. Brian noted: ‘Well, I can really sense that many have reached their limits.’ He wondered ‘if this is a healthy attitude [. . .] that you prefer to stay in the office until 2 am to keep the deadline’.

Note how Brian speaks of ‘preferring’ to stay in the office, suggesting that accountants still made their own decisions here. Yet, in contradistinction to their self-disciplined behaviour in terms of following a carefully planned blueprint, they now had to deviate from this plan and stay longer hours in the office than planned if they still wanted to meet the deadlines. Hence, their framing of such behaviour as still being their own choice (‘prefer to stay’) should be read as a ‘forced choice’, in the sense that corporate management moves away from a governing to a more coercive, disciplining form of power (Foucault, 1977).

#### Compromising other objectives

Whenever disruptions could not be compensated by increasing work intensity, accountants’ only option at this point was to neglect other objectives deemed important. Again, this became most noticeable in how accountants dealt with stretches that were communicated beyond the official schedule.

As outlined earlier, accountants considered it important to have thorough discussions with operational managers about how to incorporate stretches. However, corporate managers were said to request stretches to be worked in immediately and ‘to submit again tomorrow’ (Andy). In such cases, accountants reported to be left no option but to follow the hierarchical directives. The common procedure then was to ‘simply reduce the [stretch] as a lump sum and [. . .] to see later how to distribute it’ (Kate). In accountants’ eyes, such an approach inevitably lowered the quality of budgets and was compared to ‘shooting oneself in the foot’ (Vincent); it would ultimately backfire on themselves: ‘Because we are the ones who get trashed for not having planned well, for not having planned accurately’ (Thomas). The following quote emphasizes accountants’ reluctance to follow corporate’s orders:

At some point you become a little bit emotionless and we just try to work the stretch in – which is not necessarily beneficial. [. . .] At some point, after the tenth stretch, we capitulate and say, ‘Okay, I will work it in.’ But then we will overshoot [the budget] next year. You don’t commit yourself that much to [the budget]. Because the [budget] you did bottom-up – you’re in it with your heart to some extent. In other words, that is your numbers that you collected, that you, hopefully, challenged [operational managers] on, that you can understand and represent. [. . .] But then at some point. . . no. . . they start to saw your bone. And then it doesn’t make any sense anymore [to argue] and you say, ‘Okay fine, we have to work this in, no matter what.’ (Vincent)

The fact that, despite their personal reluctance, accountants nevertheless followed this approach signals their perceived helplessness and reveals the true power relations in the firm. This is also aptly summarized in Arnold’s statement: ‘Well, I have to submit the budget. So, I have to reduce business partnering’ (Arnold).^8^

### Temporal self-discipline, entrainment and dealing with disruptions

**Figure 2.**
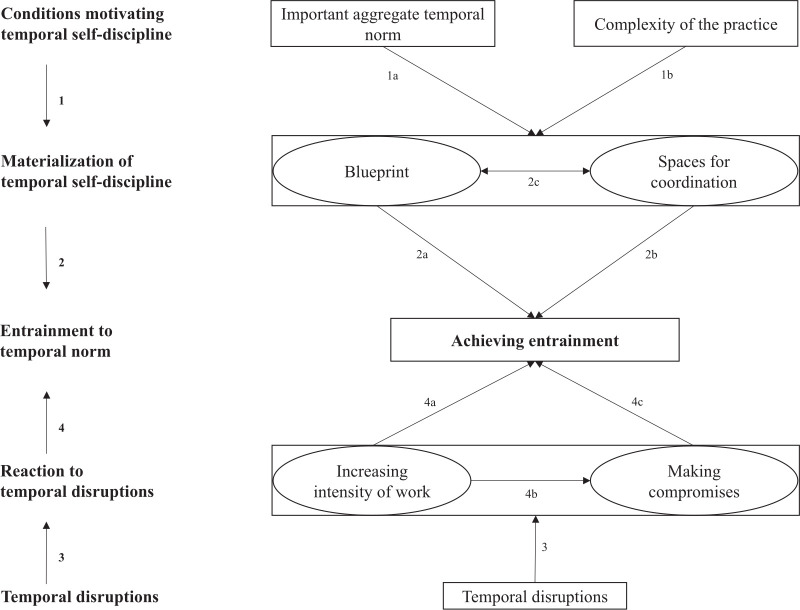
Dynamics of temporal self-discipline.

The empirical findings described above demonstrate how certain conditions facilitate the emergence of temporal self-discipline regarding a particular practice (i.e. budgeting) and how such self-discipline can become disrupted through interventions from outside. Figure 2 visualizes the observed dynamics in the form of a process model (the numbers used in the text correspond to those in Figure 2).

We put (successful) entrainment (Ancona & Chong, 1996) at the center of the model, as this is the goal towards which accountants’ efforts were directed. The upper part of the model represents the ‘controlled’ achievement of such entrainment by means of temporal self-discipline. We identified two conditions, i.e. an *important aggregate temporal norm* (1a) and the relative *complexity* of the practice (1b), which facilitated the emergence of temporal self-discipline. On the one hand, self-discipline was required because deadlines specified uncontestable goals, which however left flexibility in how to achieve them. On the other hand, the practice was complex in terms of consisting of different steps and involving different actors, thus requiring careful pre-planning.

Such self-discipline materialized in two forms in our setting, namely in a *blueprint version of the practice* outlining its ideal performance (2a) and in *spaces for coordination* to enforce this blueprint and possibly adapt it (2b). While the blueprint provided a script against which accountants could surveil their own performance, the spaces were used in order to enforce the blueprint and to make small adjustments to it over the course of the process (2c).

We refer to the result of these efforts as ‘controlled’ entrainment because, if entrainment happens by virtue of one’s temporal self-discipline, it goes along with a feeling of being in control over the process. This implies confidence about meeting the deadlines (i.e. entraining to the temporal norm) while also fulfilling other objectives deemed important (i.e. producing an accurate budget and keeping the intensity of work at a reasonable level). To be sure, controlled entrainment is not achieved ‘easily’. It requires considerable effort in the form of temporal self-discipline.

While controlled entrainment was the aspiration for accountants and the driving force behind their self-discipline, they actually had only partial control over the process. The lower part of our model shows how temporal disruptions required accountants to undertake additional efforts to achieve entrainment (3). Here, actors move from a ‘controlled’ form of entrainment ‘into a mode of reacting and firefighting’, making it impossible ‘to do something proactively’ (Julia). This increased accountants’ sense of discomfort, not only because they had to compromise important parts of their job (business partnering) (4b) and work longer hours to meet deadlines (4a), but also because they felt they were losing control over ‘their’ process. Corporate management’s interventions disrupted accountants’ self-discipline and this was seen as *undisciplined* behaviour on the part of corporate managers, in the sense that these managers neither adhered to their own temporal structures (corporate planning calendar) nor respected accountants’ self-created temporal structures. From the perspective of corporate management, such interventions were certainly deemed necessary, and may well have been from an observer’s point of view. However, accountants interpreted them as undisciplined behaviour precisely because they themselves had invested so much self-discipline in the process, which now turned out to be insufficient in the sense that additional effort was required. Hence, in the lower part of our model, accountants’ activities were no longer guided by their own temporal self-discipline, but by disciplinary interventions from above. These interventions reminded accountants of the hierarchical power structures in the organization and the fact that their autonomy as ‘process owners’ was a fragile one (Foucault, 1982; Miller & Rose, 1990).

## Discussion

Our empirical analysis has highlighted the role of temporal self-discipline as a particular form of temporal structuring. In the following, we discuss, first, how temporal self-discipline compares to other forms of temporal structuring and, second, how it relates to other expressions of self-discipline. We also comment on the limitations of our work.

### Temporal self-discipline and other forms of temporal structuring

Temporal self-discipline is a particular type of temporal structuring whereby actors impose upon themselves a detailed temporal plan (‘blueprint’) and use ‘spaces of coordination’ to monitor their performance according to this plan. In our case, such self-discipline is visible both in accountants’ behaviour – the efforts of crafting the plan and enacting it – and in their identification with the role as ‘process owners’. In other words, temporal self-discipline is both about actors’ conduct and about their identity as time-managing selves (Foucault, 1982).

Temporal self-discipline emerges, first, when the focal practice has a certain degree of complexity. As long as one engages in a small number of straightforward tasks, the need to carefully work out detailed plans seems limited and decisions can often be made with short lead time. Temporal self-discipline, in contrast, emerges when complying with an important temporal demand (e.g. a deadline) is not ensured unless one carefully envisions (all of) the required tasks and the time they need, so as to see whether everything ‘adds up’. This includes coordinating the participation of different actors and taking into account other objectives that influence the time that needs to be reckoned with for a particular task. There are, to be sure, many instances where actors pre-plan future activities by defining the sequence and timing of these activities (Janicik & Bartel, 2003; Turner & Rindova, 2018). In the case of temporal self-discipline, this pre-planning however assumes not only a particularly detailed form; it is also regarded as indispensable and as requiring particular effort and dedication, i.e. discipline, to turn it into reality.

Second, temporal self-discipline can only emerge when actors face both a strong constraint *and* a high degree of autonomy. They are constrained by having to comply with an important temporal norm, which motivates actors to engage in self-discipline in the first place. At the same time, actors have a high degree of temporal autonomy for how to comply with the important temporal norm. This is what distinguishes temporal self-discipline from settings where employees are more directly disciplined and lack temporal autonomy; for instance, when following production schedules (Taylor, 1911) or when being disciplined through mandatory time-recording routines (Anderson-Gough et al., 2001; Brown & Lewis, 2011). While in the case of temporal self-discipline, the temporal norm to which to entrain to is hierarchically imposed, the responsibility for coming up with the details of achieving entrainment is shifted towards the focal actors. Such a combination of constraint and freedom is typical for a governing form of power (Foucault, 1982) and results in actors feeling responsible for making the right decisions.

Temporal self-discipline also differs from settings where actors are driven by uncertain external events, which substantially limit their temporal autonomy for crafting detailed plans in advance. For instance, prior studies on firefighters (Geiger et al., 2021), project managers (Kremser & Blagoev, 2021) or newsmakers (Patriotta & Gruber, 2015) emphasize that these actors cannot rely on fixed time schedules, but need to learn to be responsive to upcoming events. Rather than disciplining themselves to follow a particular course of action, actors in these settings are, to an important extent, *driven* by repeated, and uncertain, external demands. While they also seek to entrain to demands in a ‘controlled way’, the extent of such control by virtue of detailed plans is much more limited than in the case of temporal self-discipline. Indeed, temporal self-discipline is fundamentally about staying in control *at all times* and making sure ‘that one is the central agent’ who drives the process (Lupu & Rokka, 2022, p. 1417), rather than being driven by someone else or making spontaneous adjustments. Such a sense of being in control at all times was constitutive for reproducing accountants’ subjectivity as ‘process owners’ who successfully manage the process towards deadlines.

Yet, precisely because temporal self-discipline relies upon a high degree of autonomy over one’s future behaviour, it also becomes particularly vulnerable to disruptions. Compared to other settings where actors routinely learn to deal with disruptions or surprises (Faraj & Xiao, 2006; Geiger et al., 2021; Patriotta & Gruber, 2015), accountants in our case built their blueprint of the practice on the condition that the process would, and should, mostly run as expected. Driven by the goal ‘to obtain an efficient machine’ (Foucault, 1977, p. 164), they included only small time buffers to deal with surprises. Accordingly, when disruptions occurred, they had to deviate from their intended course of action. In a sense, their self-disciplined approach had created a temporal corset that made them particularly vulnerable to disruptions.

This observation adds to our understanding of the temporal ‘autonomy paradox’ (Mazmanian et al., 2013). Prior literature shows that when actors formally possess temporal flexibility, they cannot always exploit it in practice, as it may be restricted by the demands of clients or the rhythms of markets to which they feel accountable (Blair-Loy, 2009; Briscoe, 2007; Evans, Kunda, & Barley, 2004). We suggest that such restriction may not only be a by-product of actors’ autonomy but its very expression. While accountants’ decision to craft and discipline themselves through self-created temporal structures was an expression of their temporal autonomy ‘in the short term’ (Mazmanian et al., 2013, p. 1337), it limited their autonomy throughout the process. Any deviation from the blueprint left them with a feeling of having lost control precisely because their sense of control was so closely linked to executing the plan as initially foreseen. Accordingly, actors may well perceive the creation of, and commitment to own, temporal structures as an expression of their autonomy; yet, within a governmentality regime, entrainment through temporal self-discipline reflects the subjection to an external power regime that it helps to reproduce and within which one’s autonomy is ultimately limited.

It is here where we also need to address the question of power. Literature on temporal structuring shows that temporal structures may become contested, with different actors using temporal structuring to stay in (or gain) control over others (Cuganesan, 2022; Granqvist & Gustafsson, 2016; McGivern et al., 2018; Reinecke & Ansari, 2014). Temporal self-discipline is an expression of being in control, but the autonomy that it builds upon is granted by others. The fragility of temporal self-discipline is therefore not only (or primarily) a fragility to contingent external events; it is a fragility-by-design, insofar as self-discipline emerges within a regime of power which it remains subject to. To the extent that self-discipline is a response to a hierarchically imposed objective or expectation, it likely remains responsive also to new hierarchical interventions, as the raison-d’être of the practice would disappear without its embeddedness into the organizational control structure. Hence, our study highlights an important connection between temporal structuring and power, where actors’ temporal structuring efforts are an expression of their own autonomy *and* their subjection to others; this implies that a sense of ‘being in control’ may easily become dominated by a sense of ‘being controlled’.

These insights are not least relevant as many contemporary organizations rely on governing forms of (temporal) control, in which individuals are provided with somewhat flexible temporal structures (de Vaujany et al., 2021), are expected to manage themselves self-responsibly (Foucault, 1982; Miller & Rose, 1990), and to treat time as a valuable resource (Blue, 2019; Feldman et al., 2020; Portschy, 2020).

### Temporal self-discipline and other forms of self-discipline

Our paper also contributes to research on self-discipline. Fundamental to all forms of self-discipline is an element of anticipation, in the sense that actors always discipline themselves in anticipation of some future state (Costas & Grey, 2014; Hoy, 2009; Portschy, 2020). This may be an aspirational state, such as the next step in the career (Grey, 1994) or it may be an event like an earnings presentation where the focal actor needs to ‘perform’ (Lambert & Pezet, 2011; Roberts et al., 2006). Self-discipline then includes efforts of working upon one’s behaviour, knowledge, speech or body image (Costas & Grey, 2014; Grey, 1994; Lambert & Pezet, 2011; Roberts et al., 2006).

Our paper adds to this literature by suggesting that self-discipline may not only be temporal in the fundamental sense of being anticipatory; it may also appear in the form of a detailed temporal structuring of one’s future behaviour. This temporal dimension should be seen as complementary to the forms of self-discipline identified in previous research. When actors assess their own behaviour against norms, impose upon themselves a particular bodily routine or subscribe to forms of self-optimization more generally, then this may potentially go along also with a detailed temporal structuring of their activities. To what extent this is the case is an empirical question however. In other settings, actors may engage in self-disciplining behaviour in a more ‘open-ended’ manner (e.g. Costas & Grey, 2014), seeking to continuously improve upon themselves without however seeing the need to establish detailed temporal scripts for their behaviour.

One issue worth examining therefore is to shed more light on different ways of working upon one’s future. We have traced the emergence of temporal self-discipline in our setting to the complexity of the practice and the aggregate temporal norm. ‘Complexity’ and ‘aggregation’ appear to different degrees, however, and this may influence the extent to which actors rely on temporal structuring of their future behaviour. For instance, the further away a deadline to be entrained to, the more unlikely it would seem that actors structure their future behaviours in detail. Similarly, we may see different emphasis on temporal structuring depending on how negotiable or flexible a temporal norm is. We therefore encourage research into the conditions that facilitate one or the other form of imagining, and working upon, one’s future activities.

Finally, temporal self-discipline also contributes to, and represents, a particular type of subjectivity. In our case, this materialized in the production of a time-conscious ‘process owner’ who is responsible for organizing the budgeting process. Again, one should see the temporal dimension here as one part of the story. While accountants more generally can be seen as a highly temporalized profession (Anderson-Gough et al., 2001), it is not *only* the detailed temporal structuring through which accountants shape their sense of self; the latter is produced also by virtue of more general norms that influence their behaviour, such as the goal to produce challenging budgets, the more general importance of timelines in the case organization or the ambition to act as business partners (Goretzki & Messner, 2019; Morales & Lambert, 2013). Still, their self-understanding as ‘process owners’ builds not least upon their time-managing abilities, as their agentic autonomy materializes particularly in their temporal structuring efforts (and achievements). This is likely to be different in other settings, where self-discipline does not have such a pronounced element of temporal structuring. Subjectivity is then primarily shaped by other types of effort or achievement, such as the creation of value (Roberts et al., 2006), the work for clients (Anderson-Gough et al., 2001) or entrepreneurial behaviour (Alawattage, Graham, & Wickramasinghe, 2019).

How the ‘time-managing self’ relates to such other subjectivities is a question worth further exploration. Most research on time and identity has focused on the association between particular occupational identities and the ‘ideal’ or ‘perfect’ worker who is constantly available and works long hours (Baker & Brewis, 2020; Feldman et al., 2020). Less is known about how time-management activities may align with, or conflict with, other types of subjectivities. On the one hand, the ‘time-managing self’ may be complementary to other subjectivities. For instance, seeing oneself as creating value is in principle compatible with managing one’s time well, insofar as good time management is an expression of efficiency and thus value (‘time is money’). Similarly, being a ‘professional’ typically includes also the expectation that one is able to manage one’s time well (Anderson-Gough et al., 2001).

On the other hand, investing in temporal structuring of one’s activities may come to be seen as a somewhat bureaucratic activity and might conflict with other subjectivities, such as the aspiration to be an entrepreneurial or innovative self. Some studies in accounting show that the routine, back-office activities of accountants can conflict with their aspiration to be a business partner (e.g. Goretzki & Messner, 2019; Morales & Lambert, 2013). Similarly, crafting elaborate plans has been contrasted with the need to stay flexible and agile (Rigby, Sutherland, & Noble, 2018).

We invite future research to examine how temporal self-discipline interacts with particular (aspirational) identities that either align with, or conflict with, the time-managing self. This also applies to how actors frame ‘disruptions’ to their own temporal plans. Indeed, the disruptions that accountants in our study complained about may in other contexts be framed as normal environmental disturbances that, for instance, an ‘agile self’ needs to be able to deal with. In other words, there is a need to look at different types of temporal subjectivities that actors may come to assume and that shape their views of themselves and their environment.

### Limitations

Our study is subject to some limitations that we wish to acknowledge. First, our analysis is mainly based on observations made during one budgeting period. While we could see some impact of accountants’ experiences with previous processes, our data did not allow us to systematically examine such ‘learning’ effects. Future research may take a more longitudinal approach and investigate how temporal structuring and self-discipline develop over time.

Second, like the site accountants, we took the input from corporate management as given and did not inquire into temporal structuring efforts at higher organizational levels. Especially in large and complex organizations, the interplay of temporal structuring at different levels would be an interesting focus of study.

Third, our analysis primarily focuses on accountants’ perspective on the budgeting practice and our data did not allow us to systematically explore the impact of interactions with other actors on the development of accountants’ temporal self-discipline. We therefore encourage future research to investigate the role of interactions for actors’ temporal self-discipline. Despite these limitations, our paper offers important insights into the dynamics of temporal self-discipline, which we hope will inspire future research.

## Conclusion

This paper examines how actors engage in *temporal self-discipline* so as to achieve entrainment of a practice to temporal norms. In so doing, it extends our understanding of the temporal dimension of organizational life (e.g. Feldman et al., 2020; Holt & Johnsen, 2019; Orlikowski & Yates, 2002). We conceptualize temporal self-discipline as actors’ conscious efforts to impose temporal structures on their own future behaviour so as to achieve ‘controlled entrainment’ to temporal norms. Our empirical findings illuminate two forms in which temporal self-discipline materializes – a blueprint and spaces for coordination, respectively – and it shows what happens when such self-discipline is disrupted. Finally, we suggest that temporal self-discipline contributes to creating a particular subjectivity as a time-managing self.

## Appendix

**Appendix 1.** Data collection process.

*Phase 1*: Observing and exploring, January 2015–September 2015	Introduction into the budgeting process and organizational setting by Thomas (Jan 8 and Jan 23 2015) Spending several days on-site to familiarize ourselves with accountants’ work and the atmosphere at *DrugSite* (Feb 5, 12, and 27 2015; Mar 30 2015; Apr 8, 9, 28, 29, and 30 2015; May 5 2015; Sep 7 2015; Oct 20 2015; May 19 2016) Participation in seven workshops of the “optimization project” (Jan 28 2015; May 7, 13, and 28 2015; Jun 8 and 9 2015; Jan 21 2016) Participation in team meetings within the accounting department (May 28 2015; Jun 11 and 22 2015; Aug 31 2015; Sep 2 2015)
*Phase 2*: Semi-structured interviews, September 2015–June 2016	Accountants at *DrugSite* (9): Peter, Thomas, Arnold (Sep 21 2015); Paula, Rachel, Kate, Andy (Sep 24 2015); Vincent (Oct 27 2015); Brian (Jun 07 2016) Accountants at *DrugDivision* (5): Robert (Jan 27 2016); Frank, Harold, Anthony (Jan 29 2017); Eric (Feb 2 2016) Operational Managers at *DrugSite* (5): Jacob, Michael (Dec 16 2015); Sebastian, Simon, Angela (Jan 07 2016) Site Heads at *DrugSite* (2): Mr White, Mr Green (Jan 27 2016)
*Phase 3*: Follow-up data collection, January 2017–September 2017	Feedback meeting with Head of the Accounting Department (Arnold) and *DrugSite*’s CFO (Mr Brown) to discuss observations (Jan 11 2017) Semi-structured interviews with the (former) process leader(s) within the accounting department (2): Julia, Ethan (Sep 15 2017)

## Notes

1. The difference between ‘disciplining’ and ‘governing’ is fluent and several authors use these concepts in a somewhat flexible way (e.g. Roberts et al., 2006).

2. Disciplinary practices may also be theorized through lenses other than the Foucauldian. In particular, several recent studies have drawn upon Lefebvre’s (2013) notion of ‘dressage’ and the role of temporal rhythms in orchestrating individual behaviour (e.g. Agar & Manolchev, 2020; Dorow & Jean, 2021).

3. All names are fictitious.

4. We could not participate in one workshop, but received the meeting minutes and discussed the workshop with the accountants afterwards.

5. The official name of the annual budget was ‘target’.

6. Accountants’ conception of ‘accuracy’ should not be adopted uncritically. An obsession with accuracy of numbers can be detrimental to their relevance and decision-usefulness. Yet, accountants’ pursuit of accuracy is key to their self-understanding and therefore crucial for their pronounced temporal self-discipline.

7. Corporate management’s cost-cuts to DrugSite’s initial budget proposal.

8. Business partnering here refers to accountants’ interactions with DrugSite’s operational managers, supporting them in their everyday activities and discussing budgeting input with them.
